# Psychometric evaluation of the GHQ-28 in a South African recidivist sample

**DOI:** 10.1371/journal.pone.0354025

**Published:** 2026-08-13

**Authors:** Thabile S. Manengela, Erhabor Sunday Idemudia, Lawrence Ejike Ugwu

**Affiliations:** 1 School of Psychosocial Health, North-West University, Mahikeng, South Africa; 2 Faculty of Humanities, North-West University, Mahikeng, South Africa; 3 Centre for Applied Psychology and Public Health Research in Africa (CAPPHRA), Africa; University of Huelva: Universidad de Huelva, SPAIN

## Abstract

This study evaluated the psychometric performance of the 28-item General Health Questionnaire (GHQ-28) in an archived South African recidivist sample. Data from 385 respondents were examined using item descriptives, corrected item-total correlations, confirmatory factor analysis, internal-consistency estimates, exploratory network analysis, and post hoc reduced-item sensitivity models; the network analysis used 327 complete cases. Among the full-scale models, the correlated four-factor model showed the best relative fit, chi-square(344) = 543.81, CFI = .944, TLI = .939, RMSEA = .042, and SRMR = .105. The elevated SRMR and several negative or weak loadings, however, indicated residual misfit and possible item-direction or scoring problems. Cronbach’s alpha was.747 for the total score and ranged from.403 to.733 across the four domains. Anxiety/Insomnia and Severe Depression performed more consistently than Somatic Symptoms and Social Dysfunction. Network results were mixed rather than uniformly convergent with the latent-variable findings. Reduced-item models produced better descriptive fit indices, but they used different item sets, were data-driven, and did not establish a validated short form. Because the original item labels and scoring key were not available for independent verification, domain-level interpretations, especially for Somatic Symptoms and Social Dysfunction, remain provisional. The results provide preliminary support for a multidimensional representation of distress while underscoring the need to verify item direction, reproduce the analyses from the archived code, and replicate the findings in independent correctional samples.

## Introduction

Psychological distress is a major concern in correctional populations, but the usefulness of a screening instrument in custody depends on whether it retains its intended measurement properties under institutional conditions. Global evidence documents substantial mental and physical morbidity among people in prison, while African studies indicate a high burden of mental-health need and limited context-sensitive assessment infrastructure [1,2].

The 28-item General Health Questionnaire (GHQ-28) is a widely used screening measure of common psychological distress. It was developed as a multidimensional version of the original GHQ and is conventionally organized into four seven-item domains: Somatic Symptoms, Anxiety/Insomnia, Social Dysfunction, and Severe Depression [3]. Binary GHQ scoring is commonly used for screening, although the instrument can also be scored using four response categories [3,4].

Widespread use does not by itself establish contextual validity. In custody, sleep, autonomy, social participation, and everyday role performance are shaped by institutional routines. Andersen and colleagues [5] therefore cautioned that imprisonment may disturb normal living and complicate interpretation of GHQ-28 responses. The same concern may not apply equally across domains, because emotionally focused items and functioning-oriented items may depend on different contextual assumptions.

The GHQ-28 structure has varied across settings and populations. A Nigerian study found a four-factor solution that differed in the clustering of somatic, anxiety, and depressive indicators [6], and work in Spanish primary care and multi-ethnic maternal samples has likewise shown that domain-level equivalence cannot be assumed [7,8]. In South Africa, de Kock and colleagues [9] found support for a four-factor representation in a Black community sample but also documented wording-related measurement concerns. That evidence does not establish equivalent functioning in correctional settings.

The gap is particularly important in African justice settings, where psychometric studies of commonly imported screening tools remain limited relative to prevalence research [2]. Correctional screening tools should therefore be examined not only at the total-score level but also at the item and domain levels [10].

The present study evaluated the item, domain, and structural performance of the GHQ-28 in an archived South African recidivist sample. It compared plausible latent models, examined internal consistency and item performance, explored symptom interconnections using network analysis, and conducted post hoc reduced-item sensitivity analyses. Because the archived dataset did not include the original item labels or a verifiable scoring key, the study also treats item-direction uncertainty as a central limitation rather than assuming that all negative loadings reflect substantive contextual effects.

## Materials and methods

### Study design

A cross-sectional psychometric design was used to evaluate the GHQ-28 at a single time point. Reporting was guided by STROBE recommendations for observational studies and COSMIN principles for measurement-property research [11–14]. The design does not provide evidence of temporal stability, longitudinal invariance, or predictive validity.

### Participants and data source

The archived analytic dataset contained 385 respondent records and 270 variables. The present analysis focused on the GHQ-28 item block (v54-v81). All records with at least partial GHQ-28 data were retained for item-level descriptive analyses; 327 respondents had complete data on all 28 GHQ items and were included in the complete-case network analysis. No completely empty GHQ response profile or duplicated full record was identified. Centre identifiers and centre-specific sample counts were not retained in the analytic archive, so clustering by correctional centre could not be evaluated.

Participants were identified in the source study as recidivists. The archived materials did not preserve a sufficiently detailed operational definition of recidivism, such as whether eligibility required a prior conviction, prior incarceration, or return to custody, and did not retain the offence-history verification procedure or a complete participant-flow record. The term recidivist is therefore retained only as the source study’s description of the sample and is not treated as an independently verified eligibility construct. The manuscript reports the verified archived sample and does not repeat the previously stated questionnaire-distribution count, which was inconsistent with the analytic dataset.

Age data were available for 384 participants (mean = 33.3 years, SD = 9.70, range = 18–65). The sample was predominantly male (94.5%). Valid percentages are reported for race and marital status because those variables contained missing data (Table 1).

**Table 1:** Demographic characteristics of the sample.

Variable	Category	n	% or descriptive value
Age (years)	Mean (SD); range	384	33.3 (9.70); 18-65
Gender	Male	364	94.5
	Female	21	5.5
Race	Black	304	93.8*
	White	7	2.2*
	Coloured	13	4.0*
Marital status	Married	31	11.0*
	Single	241	85.5*
	Divorced	5	1.8*
	Widowed	5	1.8*

* Valid percentages are reported for race and marital status because these variables contained missing data.

### Procedure

Recruitment occurred from 15 January to 12 March 2018 across eight correctional centres. The source study used site-based voluntary recruitment, which is best characterized as non-probability sampling. Written permission was obtained from North-West University and the Department of Correctional Services. The researcher coordinated access with regional and area commissioners and complied with site security procedures. Centre-level recruitment totals were not preserved, preventing assessment of centre-specific participation or clustering.

A research assistant at each centre helped identify individuals willing to hear about the study. The study purpose was explained, written informed consent was obtained, and participants were given time to complete the English-language questionnaire. Assistance was limited to reading or clarifying wording and was not intended to direct responses. Participants were informed that refusal or withdrawal would not affect correctional status, privileges, services, parole-related processes, or relationships with correctional authorities. No incentives were provided. The source documentation recorded a minimum target of 362 participants, but the exact numbers approached, distributed, returned, and excluded could not be verified from the archived materials and are therefore not reported.

### Ethical considerations

Ethical clearance was granted by the North-West University research ethics committee identified in the archived approval as HRREC (Mafikeng Campus; NWU-HS-2017–0173). Institutional permission was obtained from the Department of Correctional Services. Participation was voluntary and based on written informed consent. Questionnaires were treated as research records rather than correctional records; analyses used de-identified data, results are reported in aggregate, and electronic data were stored on a password-protected computer.

### Measure and scoring

Psychological distress was assessed with the GHQ-28 [3]. The instrument is conventionally divided into Somatic Symptoms, Anxiety/Insomnia, Social Dysfunction, and Severe Depression, with seven items per domain. In the archived data, the items corresponded to variables v54-v81.

The archived item variables were dichotomous and contained observed codes of 1 and 2. The working analysis recoded these values to 0 and 1 and treated the higher recoded value as symptom endorsement. Binary scoring is consistent with the GHQ screening tradition, but dichotomization reduces response variability and may attenuate reliability, factor loadings, and sensitivity to symptom severity relative to four-category scoring [3,4].

The original item labels, item wording, and scoring key were not available in the supplied archive. Therefore, the direction of positively and negatively worded items could not be independently verified. The negative loadings observed in the Somatic Symptoms and Social Dysfunction domains may consequently reflect coding-direction error, wording effects, contextual instability, or a combination of these factors. Items were not reversed post hoc to improve model fit. All substantive interpretations of these domains are explicitly provisional pending verification and reanalysis from the original codebook.

Subscale scores were calculated as sums of the seven items in each domain, and the total score was calculated as the sum of all 28 items. A summed score was treated as missing when any component item was missing.

### Data preparation and missing data

Data preparation included verification of item availability, inspection of response distributions, item-level missingness, completely empty GHQ profiles, and duplicated records. Item-level analyses used available observations. Summed-score reliability estimates used complete data for the relevant scale, and the network analysis used 327 complete GHQ cases.

The supplied manuscript output did not preserve the exact lavaan missing-data argument or the analytic sample size for each CFA model. These details should be verified against the final public R script before resubmission. The present report therefore does not claim a reconstructed CFA sample size that cannot be confirmed from the available files.

### Structural validity

Confirmatory factor analysis was conducted in lavaan using the weighted least squares mean and variance adjusted estimator (WLSMV). The binary items were specified as ordered indicators, so the models used thresholds and the underlying tetrachoric association structure. Three models were specified: a one-factor model; a correlated four-factor model corresponding to the conventional GHQ-28 domains; and a higher-order model in which the four first-order domains loaded on a general distress factor.

Latent variances were standardized (std.lv = TRUE). Model evaluation used chi-square, the Comparative Fit Index (CFI), Tucker-Lewis Index (TLI), Root Mean Square Error of Approximation (RMSEA), and Standardized Root Mean Square Residual (SRMR). Model selection considered relative fit, parsimony, theoretical plausibility, and interpretability. Conventional fit thresholds were treated as descriptive guides rather than automatic decision rules.

### Reliability and item evaluation

Internal consistency was summarized using raw Cronbach’s alpha, standardized alpha, and the average inter-item correlation for the total scale and each domain. Model-based omega values were removed from the final report because archived outputs from different procedures were internally inconsistent and could not be reconciled without rerunning the analysis. This prevents overinterpretation of potentially non-comparable omega estimates.

Items were evaluated using corrected item-total correlations, standardized CFA loadings, and exploratory network strength. Values below approximately.20 for corrected item-total correlations, below approximately.40 for standardized loadings, or near zero for network strength were treated only as descriptive warning indicators. No single threshold was used as an automatic deletion rule.

### Exploratory network analysis

An Ising model was estimated in bootnet using default = “IsingFit” for the 327 complete GHQ cases. Strength, expected influence, and betweenness were extracted. The full-network centrality plot is provided in S1 Fig. For the exploratory Reduced B network, the item network, strength centrality plot, bootstrap edge-weight intervals, and bootstrap strength-difference plot are provided in in S2-S5 Figs. A reproducible case-dropping bootstrap analysis and correlation-stability coefficient were not available. The centrality and bootstrap findings were therefore interpreted descriptively and were not used as the sole basis for item-retention or deletion decisions.

### Exploratory reduced-item sensitivity analyses

Three post hoc reduced-item models and one three-factor model were examined as sensitivity analyses. Reduced A omitted v54; Reduced B omitted v54, v68, v70, and v74; Reduced C omitted v54, v55, v68, v70, v72, v73, and v74; and the three-factor model omitted the complete Social Dysfunction block. These analyses were not preregistered and were not intended to validate a new short form.

Because the reduced models contained different observed indicators, their fit indices are descriptive and are not treated as direct tests of superiority over the full model. The archived output flagged inadmissible latent relations in Reduced C, but it did not preserve the relevant convergence flag, factor-correlation matrix, residual variances, or other diagnostic information needed to identify the specific source of inadmissibility. Accordingly, Reduced C was not interpreted and no reduced solution was selected as a final instrument.

### Software

Analyses were conducted in R using tidyverse, janitor, and readr for data preparation; psych for descriptive statistics and internal consistency; lavaan for CFA; and qgraph and bootnet for network estimation and visualization. Before public archiving, the repository package should be checked against the ethics approval and consent conditions and screened for indirect identifiers. The final record should contain the de-identified analytic data, a variable dictionary and scoring key, the complete R script, package and session information, and a README linking each manuscript table and figure to its generating code.

## Results

### Item-level performance

Item-level missingness ranged from 0 to 11 responses, and the number of non-missing observations per item ranged from 374 to 385. Endorsement proportions ranged from.123 for v68 to.653 for v77. Corrected item-total correlations were heterogeneous (Table 2). The weakest coefficients were observed for v54, v74, v73, v72, v70, and v68, whereas v79, v80, v66, and v64 had the strongest coefficients. These patterns should not be interpreted independently of the unresolved item-direction issue.

**Table 2:** GHQ-28 item descriptives and corrected item-total correlations.

Item	n	Endorsement	Variance	Corrected item-total r
v54	384	0.250	0.188	−0.228
v55	383	0.363	0.232	0.156
v56	374	0.521	0.250	0.279
v57	380	0.445	0.248	0.338
v58	382	0.351	0.228	0.393
v59	381	0.391	0.239	0.393
v60	382	0.513	0.250	0.307
v61	384	0.339	0.225	0.386
v62	383	0.452	0.248	0.328
v63	380	0.463	0.249	0.394
v64	380	0.482	0.250	0.412
v65	381	0.559	0.247	0.403
v66	385	0.475	0.250	0.421
v67	379	0.628	0.234	0.383
v68	381	0.123	0.108	0.016
v69	383	0.399	0.240	0.306
v70	380	0.174	0.144	0.016
v71	379	0.166	0.139	0.035
v72	382	0.272	0.199	−0.006
v73	382	0.209	0.166	−0.014
v74	381	0.186	0.152	−0.085
v75	382	0.607	0.238	0.320
v76	381	0.638	0.231	0.389
v77	378	0.653	0.227	0.329
v78	381	0.467	0.250	0.285
v79	381	0.444	0.248	0.432
v80	382	0.581	0.244	0.426
v81	383	0.548	0.248	0.352

### Confirmatory factor analysis

The correlated four-factor model had the best relative fit among the three full-scale models (Table 3). Its CFI, TLI, and RMSEA were stronger than those of the one-factor and higher-order models, but the SRMR remained elevated at.105. The model is therefore described as the best-fitting candidate rather than as unequivocally well fitting.

**Table 3:** Fit indices for the full GHQ-28 CFA models.

Model	chi-square	df	CFI	TLI	RMSEA	SRMR
One-factor	756.08	350	.886	.877	.060	.118
Correlated four-factor	543.81	344	.944	.939	.042	.105
Higher-order	566.20	346	.938	.933	.044	.107

Standardized loadings in the Anxiety/Insomnia and Severe Depression domains were generally moderate to strong. The Somatic Symptoms domain contained several negative loadings, and Social Dysfunction included weak, non-significant, and negative loadings (Table 4). Given the absence of a verifiable item-direction key, these signs cannot be interpreted as substantive protective effects and may indicate a coding or wording artifact.

**Table 4:** Standardized loadings for the correlated four-factor model.

Factor	Item	Loading	p
Somatic	v54	.561	<.001
Somatic	v55	−.241	.004
Somatic	v56	−.559	<.001
Somatic	v57	−.655	<.001
Somatic	v58	−.723	<.001
Somatic	v59	−.688	<.001
Somatic	v60	−.553	<.001
Anxiety/Insomnia	v61	.639	<.001
Anxiety/Insomnia	v62	.526	<.001
Anxiety/Insomnia	v63	.573	<.001
Anxiety/Insomnia	v64	.659	<.001
Anxiety/Insomnia	v65	.649	<.001
Anxiety/Insomnia	v66	.730	<.001
Anxiety/Insomnia	v67	.608	<.001
Social Dysfunction	v68	.254	.085
Social Dysfunction	v69	−.747	<.001
Social Dysfunction	v70	.095	.454
Social Dysfunction	v71	.161	.187
Social Dysfunction	v72	.244	.015
Social Dysfunction	v73	.321	.003
Social Dysfunction	v74	.492	<.001
Severe Depression	v75	.564	<.001
Severe Depression	v76	.780	<.001
Severe Depression	v77	.700	<.001
Severe Depression	v78	.539	<.001
Severe Depression	v79	.689	<.001
Severe Depression	v80	.770	<.001
Severe Depression	v81	.696	<.001

Latent factor correlations for the correlated four-factor model are reported in Supplementary S1 Table. Their signs and magnitudes should be interpreted cautiously because the item-direction key was unavailable and the orientation of the Somatic Symptoms and Social Dysfunction factors could not be independently verified.

### Internal consistency

Cronbach’s alpha was.747 for the total score. Anxiety/Insomnia and Severe Depression had the strongest domain estimates, whereas Somatic Symptoms and Social Dysfunction had low alpha values (Table 5). These estimates reflect the working binary scoring and may be distorted if item direction was not aligned.

**Table 5:** Internal-consistency indices for the full GHQ-28.

Scale	Raw alpha	Standardized alpha	Average inter-item r
GHQ-28 total	.747	.726	.086
Somatic Symptoms	.411	.385	.082
Anxiety/Insomnia	.684	.684	.237
Social Dysfunction	.403	.433	.098
Severe Depression	.733	.734	.282

### Exploratory network findings

**Fig 1:**
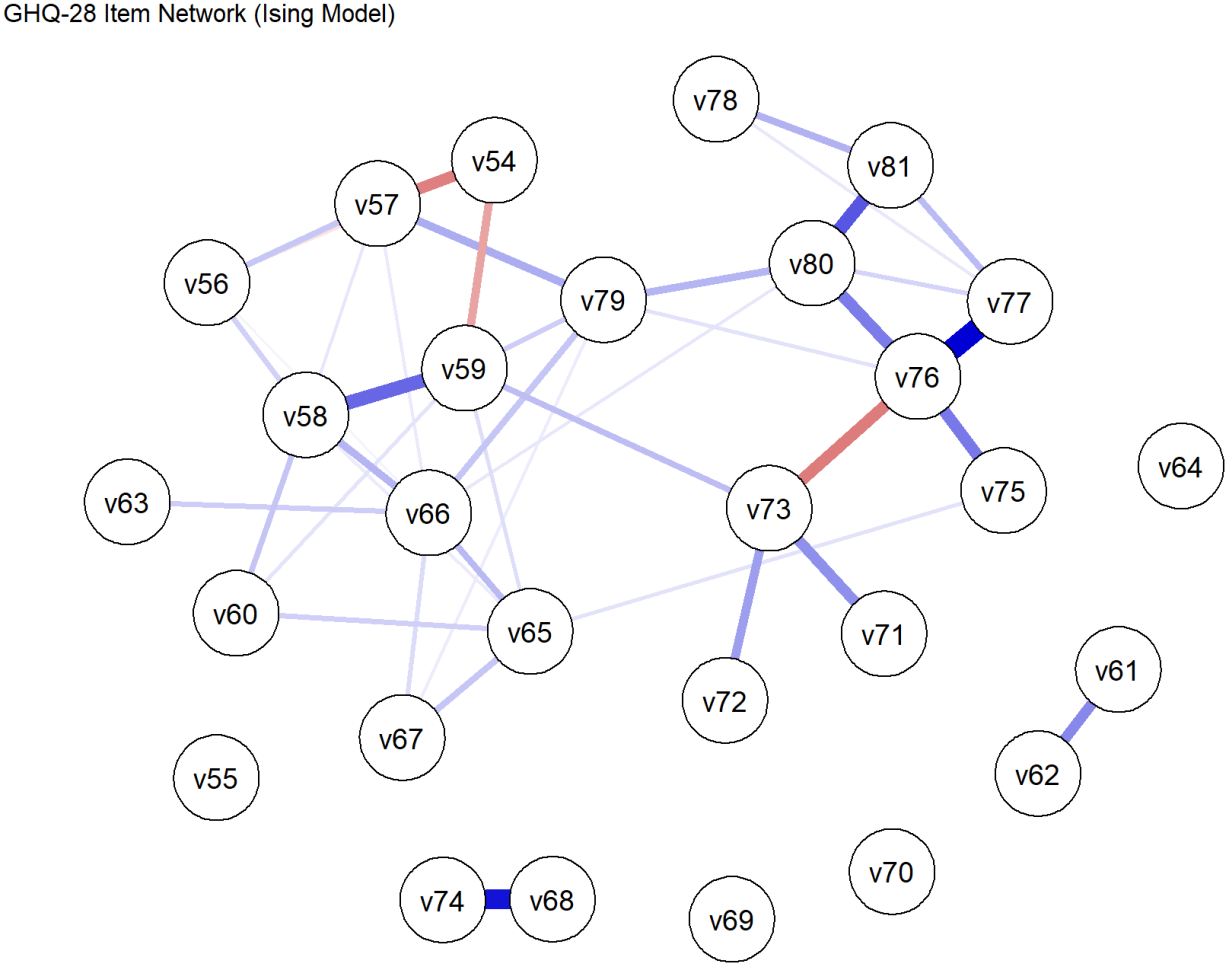
Full GHQ-28 item network (Ising model). Blue edges indicate positive conditional associations and red edges indicate negative conditional associations. Thicker edges represent stronger associations. The figure is supplied as a separate file, as required for journal submission.

The Ising network was estimated from 327 complete cases and is presented in Fig 1. Some findings converged with the item-total and CFA results, such as zero strength for v55 and v70. Other findings diverged: v73 had a weak corrected item-total correlation and modest loading but was among the most central network nodes, while v64 had a strong corrected item-total correlation and loading but zero network strength. The network therefore provided mixed, not uniformly convergent, evidence (Table 6). The complete full-network centrality profile is presented in S1 Fig. Because a case-dropping bootstrap analysis and correlation-stability coefficient were not available, the centrality findings were interpreted cautiously and were not used as the sole basis for item-retention decisions.

**Table 6:** Selected network centrality values for full GHQ-28 items.

Item	Strength	Expected influence	Betweenness
v76	4.472	2.748	52
v80	2.893	2.893	47
v59	2.754	1.554	55
v73	2.661	.937	59
v77	2.555	2.555	0
v58	2.456	2.456	37
v66	2.244	2.244	32
v79	2.045	2.045	34
v55	0.000	0.000	0
v64	0.000	0.000	0
v69	0.000	0.000	0
v70	0.000	0.000	0

### Exploratory sensitivity models

Reduced-item and no-Social-Dysfunction models produced numerically stronger fit indices than the full model (Table 7). These values are descriptive because the models were fitted to different item sets and cannot be treated as direct evidence that a reduced instrument is superior. Reduced C was not interpreted because the archived output flagged an inadmissible solution but did not retain the convergence and parameter diagnostics required to determine its exact cause.

**Table 7:** Descriptive fit indices for full and exploratory reduced models.

Model	Items	chi-square	df	CFI	TLI	RMSEA	SRMR	Alpha
Full four-factor	28	543.81	344	.944	.939	.042	.105	.747
Reduced A	27	506.00	318	.944	.938	.043	.104	.766
Reduced B	24	365.00	246	.964	.959	.038	.094	.788
Reduced C	21	225.00	183	.987	.985	.026	.079	.811
Three-factor, no Social Dysfunction	21	229.00	186	.986	.985	.026	.079	–

*Models contain different observed indicators; fit indices are descriptive and should not be compared as formal nested-model tests.*

The Reduced B item network and strength centrality plot are presented in S2 and S3 Figs, respectively. Detailed standardized loadings and internal-consistency estimates for Reduced B are reported in S2 Table Table in S1 File. Archived bootstrap edge-weight intervals and strength-difference results are presented in S4 and S5. These partial bootstrap diagnostics do not provide a case-dropping correlation-stability coefficient and should not be interpreted as establishing network stability or validating Reduced B as a short form.

An audit of the candidate item decisions showed that the deletion rationale was not consistently supported across indices (Table 8). In particular, v71 met two warning criteria but was retained, v73 was central in the network despite weak latent and item-total indices, and v74 had the strongest positive Social Dysfunction loading but was deleted in two reduced models. No reduced form was therefore retained as a validated alternative.

**Table 8:** Exploratory item-decision audit.

Item	Corrected item-total r	Loading	Network strength
v54	−.228	.561	NR
v55	.156	−.241	0.000
v68	.016	.254	NR
v70	.016	.095	0.000
v71	.035	.161	NR
v72	−.006	.244	NR
v73	−.014	.321	2.661
v74	−.085	.492	NR

*NR = not reported in the archived network-centrality output.*

## Discussion

The correlated four-factor model provided the strongest relative representation of the GHQ-28 among the full-scale models, but the findings do not justify an unqualified claim of structural adequacy. The elevated SRMR, mixed loading signs, weak domain alphas, and unresolved item-direction issue indicate that the model requires verification before strong domain-level conclusions are drawn.

### Item direction and contextual interpretation

The most important interpretive issue is that the original item labels and scoring key were not available. The negative loadings in Somatic Symptoms and Social Dysfunction closely resemble the kind of pattern that can arise when positively and negatively worded items are not aligned. Wording effects have previously been documented in a Black South African GHQ-28 sample [9]. Therefore, contextual distortion caused by incarceration is only one plausible explanation; coding direction and wording effects are equally important alternatives.

The Social Dysfunction domain showed low alpha and several weak loadings, but this should not be taken as proof that the construct is invalid in custody. If item direction is verified and the pattern persists, institutional restrictions on autonomy, routine, and ordinary role performance could contribute to contextual distortion, as suggested by prior prison validation work [5]. Until that verification is completed, the present results support caution rather than a definitive contextual explanation.

### Domain-level performance

Anxiety/Insomnia and Severe Depression were more internally consistent and had more coherent loadings than the other domains. Somatic Symptoms and Social Dysfunction were less stable. This pattern may reflect genuine differences in transportability, but it may also reflect wording and scoring artifacts. Accordingly, the emotional symptom domains appear more robust in the present working analysis, but the contrast should be replicated after verified scoring.

### Reduced models and network analysis

The reduced-item analyses did not validate a short form. Fit improved numerically as items were removed, but the models used different indicators, the deletion rule was inconsistently applied, and the most aggressive model was inadmissible for an undetermined reason. Similarly, the network analysis produced both convergent and discordant findings. The supplementary material preserves partial Reduced B bootstrap diagnostics, but a case-dropping correlation-stability coefficient was unavailable. These analyses are best treated as hypothesis-generating tools for a future preregistered study rather than as evidence for item deletion or a preferred short form.

### Implications

Researchers should avoid assuming that all GHQ-28 domains are equally interpretable in correctional settings. Total-score and domain-score decisions should be supported by verified scoring, transparent missing-data handling, and replicated structural evidence. Correctional applications may eventually require adapted functioning items that distinguish psychological impairment from institutionally imposed restrictions, but such adaptation should follow item-level qualitative work and independent validation.

### Limitations

The study was cross-sectional and based on a single archived dataset. The sample was predominantly male and drawn from one South African correctional population, limiting generalizability. Although recruitment occurred across eight centres, centre-specific counts and identifiers were unavailable, so clustering and site heterogeneity could not be assessed. The archived materials did not preserve a complete participant-flow record, an independently verifiable operational definition of recidivism, the original GHQ item labels and scoring key, or the exact CFA missing-data argument. Questionnaire assistance may have introduced response bias. Binary scoring reduced response variability. Only partial Reduced B bootstrap diagnostics were preserved; a reproducible case-dropping bootstrap analysis and correlation-stability coefficient were unavailable. The exact source of the Reduced C inadmissibility could not be identified, and the reduced-item analyses were post hoc and data-driven. These limitations mean that the reported numerical results should be considered preliminary until the analysis is reproduced from a verified codebook and complete script.

### Future directions

The highest priority is to verify the original item wording and direction, reconstruct the scoring key, and rerun all descriptives, reliability estimates, CFA models, network analyses, and sensitivity models. The rerun should include full-network and reduced-network bootstrap edge-weight accuracy, strength-difference, and case-dropping stability analyses and should preserve complete convergence logs, parameter diagnostics, session information, and model-specific sample sizes. Future studies should report participant flow and centre-level recruitment, account for clustering where appropriate, compare binary and four-category scoring, test measurement invariance across correctional groups and sex, and evaluate temporal stability and predictive validity. Qualitative cognitive interviewing could help determine whether Social Dysfunction items capture psychological impairment or institutional restrictions.

## Conclusion

The GHQ-28 showed preliminary evidence of a multidimensional structure in this South African recidivist sample, with the correlated four-factor model fitting better than the tested alternatives. Nevertheless, elevated residual misfit, low reliability in two domains, mixed network evidence, and unresolved item-direction uncertainty prevent definitive conclusions about domain transportability. The findings support cautious use and a transparent reanalysis rather than validation of a new short form or a firm claim that Social Dysfunction is uniquely distorted by custody.

## Supporting information

S1 Fig Full GHQ-28 centrality plot. Centrality plot for the full GHQ-28 Ising network. The figure is provided as supplementary descriptive evidence and should be interpreted cautiously because a case-dropping bootstrap analysis and correlation-stability coefficient were not available.
(PNG)

S2 Fig Reduced B GHQ item network (Ising model). Item network for the exploratory Reduced B solution after omission of v54, v68, v70, and v74. The model was examined as a post hoc sensitivity analysis and should not be interpreted as a validated short form.
(PNG)

S3 Fig Reduced B centrality plot. Strength centrality plot for the Reduced B network. Higher values indicate stronger connectivity to other retained items in the reduced exploratory network.
(PNG)

S4 Fig Reduced B bootstrap edge-weight intervals. Bootstrap edge-weight intervals for the Reduced B network. This partial diagnostic is included for technical transparency and should be read as exploratory because a complete case-dropping stability analysis and correlation-stability coefficient were unavailable.
(PNG)

S5 Fig Reduced B bootstrap strength-difference plot. Bootstrap-based strength-difference plot for the Reduced B network. This diagnostic is presented as supplementary information rather than as main-text inferential evidence.
(PNG)

S1 File Supplementary tables supporting the GHQ-28 psychometric analyses. This file contains S1 Table, latent factor correlations for the preferred four-factor model, and S2 Table, Reduced B loadings and subscale reliability.
(DOCX)

## References

[1] Favril L, Rich JD, Hard J, Fazel S. Mental and physical health morbidity among people in prisons: an umbrella review. Lancet Public Health. 2024;9(4):e250–60. doi: 10.1016/S2468-2667(24)00023-9 38553144 PMC11652378

[2] Lovett A, Kwon HR, Kidia K, Machando D, Crooks M, Fricchione G, et al. Mental health of people detained within the justice system in Africa: systematic review and meta-analysis. Int J Ment Health Syst. 2019;13:31. doi: 10.1186/s13033-019-0273-z 31080500 PMC6501291

[3] Goldberg DP, Hillier VF. A scaled version of the General Health Questionnaire. Psychol Med. 1979;9(1):139–45. doi: 10.1017/s0033291700021644 424481

[4] Chipimo PJ, Fylkesnes K. Comparative validity of screening instruments for mental distress in zambia. Clin Pract Epidemiol Ment Health. 2010;6:4–15. doi: 10.2174/1745017901006010004 20498698 PMC2858522

[5] Andersen HS, Sestoft D, Lillebaek T, Gabrielsen G, Hemmingsen R. Validity of the General Health Questionnaire (GHQ-28) in a prison population: data from a randomized sample of prisoners on remand. Int J Law Psychiatry. 2002;25(6):573–80. doi: 10.1016/s0160-2527(01)00085-1 12414023

[6] Aderibigbe YA, Riley W, Lewin T, Gureje O. Factor structure of the 28-item general health questionnaire in a sample of antenatal women. Int J Psychiatry Med. 1996;26(3):263–9. doi: 10.2190/3XAV-M1BC-DA2B-DCMF 8976467

[7] Molina JD, Andrade-Rosa C, Gonzalez-Parra S, Blasco-Fontecilla H, Real MA, Pintor L. The GHQ-28 factor structure in Spanish primary care. Int J Psychiatry Med. 2006;36(4):397–407.

[8] Prady SL, Miles JNV, Pickett KE, Fairley L, Bloor K, Gilbody S. Cross-cultural measurement equivalence of the GHQ-28 in a multi-ethnic maternal sample. J Affect Disord. 2013;147(1–3):272–80.

[9] de Kock FS, Görgens-Ekermans G, Dhladhla TJ. A confirmatory factor analysis of the General Health Questionnaire-28 in a Black South African sample. J Health Psychol. 2014;19(10):1222–31. doi: 10.1177/1359105313488972 23740263

[10] Martin MS, Colman I, Simpson AIF, McKenzie K. Mental health screening tools in correctional institutions: a systematic review. BMC Psychiatry. 2013;13:275. doi: 10.1186/1471-244X-13-275 24168162 PMC4231452

[11] Mokkink LB, Terwee CB, Patrick DL, Alonso J, Stratford PW, Knol DL, et al. The COSMIN checklist for assessing the methodological quality of studies on measurement properties of health status measurement instruments: an international Delphi study. Qual Life Res. 2010;19(4):539–49. doi: 10.1007/s11136-010-9606-8 20169472 PMC2852520

[12] von Elm E, Altman DG, Egger M, Pocock SJ, Gøtzsche PC, Vandenbroucke JP, et al. The strengthening the reporting of observational studies in epidemiology (strobe) statement: guidelines for reporting observational studies. PLoS Med. 2007;4(10):e296. doi: 10.1371/journal.pmed.0040296 17941714 PMC2020495

[13] Vieira APTDA, Coelho GE, da Silva AC, Madeira N. Assessment of the factor structure and reliability of the GHQ-28 in a Portuguese non-clinical sample. Eur J Psychol Assess. 2011;27(4):258–63.

[14] Weyerer S, Elton M, Diallina M, Fichter MM. The principal component structure of the General Health Questionnaire among Greek and Turkish adolescents. Eur Arch Psychiatry Neurol Sci. 1986;236(2):75–82. doi: 10.1007/BF00454015 3792410

