# Supplementary material for: Psychometric evaluation of the GHQ-28 in a South African recidivist sample
Source: PLoS One. 2026 Aug 13;21(8):e0354025. doi: 10.1371/journal.pone.0354025 (PMC13472388; doi:10.1371/journal.pone.0354025)
Supplement: Supplementary file 1 [file pone.0354025.s006.docx]

S1 File

Supplementary tables supporting the GHQ-28 psychometric analyses

This supporting-information file contains supplementary tables cited in the manuscript.

**Supplementary Tables**

*Additional tabular results referenced in the main manuscript*

**Supplementary Table S1. Latent factor correlations for the preferred four-factor model**

This table complements the CFA results in the main manuscript. The signs and magnitudes of the correlations should be interpreted cautiously because the original item-direction key was unavailable and latent-factor orientation could not be independently verified.

| **Factor** | **Somatic** | **Anxiety** | **Social** | **Depression** |
| --- | --- | --- | --- | --- |
| Somatic | 1.000 |  |  |  |
| Anxiety | -0.757 | 1.000 |  |  |
| Social | 0.288 | -0.496 | 1.000 |  |
| Depression | -0.465 | 0.657 | -0.572 | 1.000 |

**Supplementary Table S2. Reduced B loadings and subscale reliability**

This table provides detailed loadings and subscale reliability estimates for the exploratory Reduced B model. It is included for transparency and does not establish a validated short form.

| Panel A. Reduced B loadings |  |  |  |
| --- | --- | --- | --- |
| Factor | Item | Loading | p |
| Somatic | v55 | .250 | .003 |
| Somatic | v56 | .531 | <.001 |
| Somatic | v57 | .598 | <.001 |
| Somatic | v58 | .733 | <.001 |
| Somatic | v59 | .662 | <.001 |
| Somatic | v60 | .553 | <.001 |
| Anxiety | v61 | .637 | <.001 |
| Anxiety | v62 | .534 | <.001 |
| Anxiety | v63 | .575 | <.001 |
| Anxiety | v64 | .649 | <.001 |
| Anxiety | v65 | .659 | <.001 |
| Anxiety | v66 | .713 | <.001 |
| Anxiety | v67 | .613 | <.001 |
| Social | v69 | .881 | <.001 |
| Social | v71 | -.137 | .259 |
| Social | v72 | -.219 | .023 |
| Social | v73 | -.270 | .011 |
| Depression | v75 | .567 | <.001 |
| Depression | v76 | .773 | <.001 |
| Depression | v77 | .698 | <.001 |
| Depression | v78 | .520 | <.001 |
| Depression | v79 | .684 | <.001 |
| Depression | v80 | .776 | <.001 |
| Depression | v81 | .708 | <.001 |

Note. The direction of the Somatic Symptoms and Social Dysfunction loadings differs from the full-model orientation. Loading signs should not be compared across models until factor orientation and the original item-scoring direction have been verified.

**Panel B. Reduced B subscale reliability**

| **Subscale** | **n items** | **Raw alpha** | **Std. alpha** | Average inter-item r |
| --- | --- | --- | --- | --- |
| Somatic | 6 | .580 | .580 | .187 |
| Anxiety | 7 | .684 | .684 | .237 |
| Social | 4 | .239 | .277 | .087 |
| Depression | 7 | .733 | .734 | .282 |

Note. Reduced B was a post hoc sensitivity model and was not retained as a validated alternative. Social Dysfunction remained weak (raw alpha = .239; standardized alpha = .277).
